# Aortic Dissection Type A in Alpine Skiers

**DOI:** 10.1155/2013/192459

**Published:** 2013-07-21

**Authors:** Thomas Schachner, Nikolaus Fischler, Julia Dumfarth, Nikolaos Bonaros, Christoph Krapf, Wolfgang Schobersberger, Michael Grimm

**Affiliations:** ^1^Innsbruck Medical University, 6020 Innsbruck, Austria; ^2^Institute for Sports Medicine, Alpine Medicine and Health Tourism, University Hospital Innsbruck and UMIT Hall, Austria

## Abstract

*Patients and Methods.* 140 patients with aortic dissection type A were admitted for cardiac surgery. Seventy-seven patients experienced their dissection in the winter season (from November to April). We analyzed cases of ascending aortic dissection associated with alpine skiing. *Results.* In 17 patients we found skiing-related aortic dissections. Skiers were taller (180 (172–200) cm versus 175 (157–191) cm, *P* = 0.008) and heavier (90 (68–125) kg versus 80 (45–110) kg, *P* = 0.002) than nonskiers. An extension of aortic dissection into the aortic arch, the descending thoracic aorta, and the abdominal aorta was found in 91%, 74%, and 69%, respectively, with no significant difference between skiers and nonskiers. Skiers experienced RCA ostium dissection requiring CABG in 17.6% while this was true for 5% of nonskiers (*P* = 0.086). Hospital mortality of skiers was 6% versus 13% in nonskiers (*P* = 0.399). The skiers live at an altitude of 170 (0–853) m.a.s.l. and experience their dissection at 1602 (1185–3105; *P* < 0.001) m.a.s.l. In 82% symptom start was during recreational skiing without any trauma. *Conclusion.* Skiing associated aortic dissection type A is usually nontraumatic. The persons affected live at low altitudes and practice an outdoor sport at unusual high altitude at cold temperatures. Postoperative outcome is good.

## 1. Background

Alpine skiing is a popular sports activity. For guests who are not acclimatized to high altitudes adaptation to the alpine climate means physical stress due to the combination of hypoxia plus unusual outdoor activity at low ambient temperatures. Skiing is known to be a complex sport with a relatively high demand of conditioning due to the typical pattern of short periods of moderate to heavy strenuous exercise and longer phases of interruption [1]. Due to these complex demands including cardiorespiratory, neuromuscular, and sensorimotor systems alpine skiing can be a challenge especially for the elder skier [2]. Additional stress for the aorta may result from highly variable increased intrathoracic pressures during carving maneuvers of the skiers, mainly during stopping of the movement. Hiratzka et al. mention in the guidelines for the diagnosis and management of patients with thoracic aortic disease that thoracic stress or trauma may result from a skiing accident but in a typical traumatic injury of the descending aorta [3]. Scarce information about skiing-associated aortic dissection is present and it is at present limited to case reports on posttraumatic descending aortic injury. Heller et al. report 4 cases of traumatic aortic rupture following skiing crashes with a mortality of one out of four [4]. The University Hospital in Innsbruck is surrounded by the Alps; thus, many skiing resorts are within our referral area. In the county of Tirol live about 714.000 permanent residents, and just in the winter season 2010/2011 5.1 million guests visited Tirol [5]. Accordingly, we found that all our patients in this study with ascending aortic dissection were recreational skiers. We aimed to analyze in detail all cases of skiing-associated ascending aortic dissection Stanford type A which were admitted to the clinic of cardiac surgery over a 10-year period. 

## 2. Patients and Methods

From the years 2001 to 2011 all patients with aortic dissection involving the ascending aorta who were admitted to our clinic of cardiac surgery were entered into a database. We retrospectively analyzed the patient records and additionally contacted the patients or their relatives and the referring physician/hospital to gain information on the place where and the circumstances how the aortic dissection happened. We defined the winter season as a period of 6 months from the beginning of November until the end of April. This is in accordance with the common definition of winter season of the tourism agencies. All skiing-related aortic dissections occurred within this time interval.

Common demographic and geographic data were collected from official boards in the different countries of origin of the patients and local governmental and touristic boards of the Tyrolean region. The height above sea level in a certain skiing resort was defined as the average between the lowest spot and the highest spot within the resort. 

For statistical analysis, the statistical software package SPSS 17.0 (SPSS, Chicago, Illinois) was used. Categorical parameters were displayed as numbers and percentages; the continuous variables were displayed as median and range. Differences between groups were calculated using the Mann-Whitney *U* test (continuous variables) or the chi-square test (categorical variables). A *P* value <0.05 was considered statistically significant.

## 3. Results

During the study period 140 patients with type A aortic dissection were admitted to our clinic of cardiac surgery. 77/140 (55%) patients were admitted during the winter seasons from the beginning of November until the end of April. During the winter season 17/77 (22.1%) of type A aortic dissections were associated with alpine skiing.

The number of persons without permanent residence within our referral area was significantly higher among skiers compared with nonskiers (16/17 (94.1%) versus 10/60 (16.7%), *P* < 0.001). 

The demographic data are displayed in Table 1. We found that patients in the skier group were significantly taller and heavier compared with nonskiers. There was, however, no difference with regard to preoperative status between the two groups. 

We found no difference between nonskiers and skiers with regard to the extension of aortic dissection into the aortic arch (91.7% versus 88.2%, *P* = 0.664), into the descending thoracic aorta (71.7% versus 82.4%, *P* = 0.375), or into the abdominal aorta (68.3% versus 70.6%, *P* = 0.859).

Intraoperatively, 3/17 (17.6%) skiers and 3/60 (5%) of nonskiers had a dissection of the right coronary ostium which required coronary bypass surgery (*P* = 0.086). There was no significant difference with regard to other intraoperative variables between the two groups (Table 2).

1 out of 17 (5.9%) skiers died in hospital due to bleeding from the descending aorta one week after the operation. Among the nonskiers hospital mortality was 13.3% (skiers versus nonskiers *P* = 0.399).

**Figure 1:**
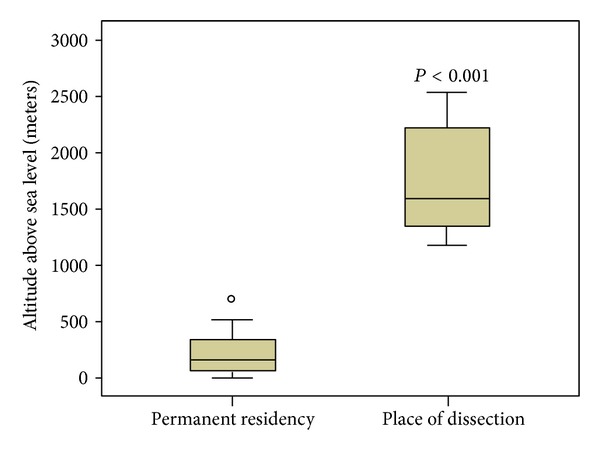
Altitude above sea level of the place of permanent residency and of the place where aortic dissection occurred in 17 skiers. Note the marker of the single Tyrolean skier among 17 skiing-associated aortic dissections type A whose place of living was at an altitude of 853 meters.

The permanent residency of skiers was at an altitude of 170 (0–853) m.a.s.l. and the place of aortic dissection was at 1602 (1185–3105) m.a.s.l. meters, *P* < 0.001. See Figure 1.

**Figure 2:**
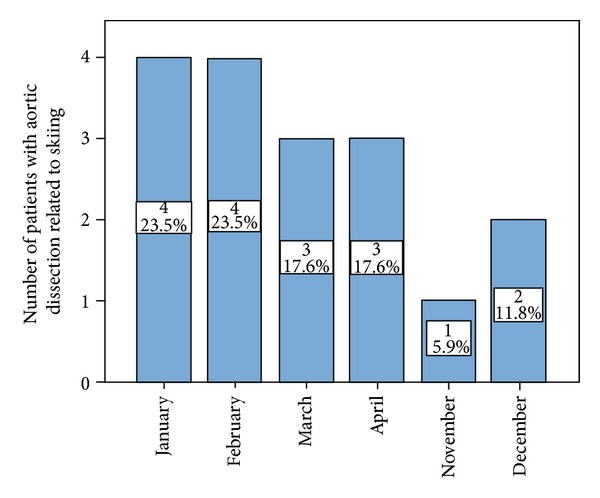
Frequency of skiing-associated aortic dissection type A among the different months during the winter season.

Among skiers 8/17 (47.1%) aortic dissections occurred in January or February (Figure 2).

All patients experienced pain predominantly in the chest. In addition 2/17 (12%) patients had abdominal pain, 1/17 (6%) had head/neck pain (due to carotid artery dissection), 1/17 (6%) patient experienced painful legs, and 2/17 (12%) patients had a syncope. 

Surprisingly, we found in 14/17 (82%) patients a start of symptoms during recreational skiing without additional trauma. Only 1 patient (6%) had a skiing accident with consequent ascending aortic dissection. One patient experienced pain after the ascent with the cable car, and another one during lunch at the skiing hut.

## 4. Discussion

Alpine skiing is a very popular sport, and many skiing resorts are situated within our referral area of moderate to high altitude. The guests who come for recreational skiing typically live at a low altitude. In our study the skiers who experienced aortic dissection lived at an altitude of 170 m.a.s.l. and had their aortic dissection at a tenfold higher altitude. Are there preexisting comorbidities among alpine skiers? A questionnaire study on more than 1400 skiers in the Austrian Alps revealed a prevalence of cardiovascular diseases (mainly hypertension) in this population of 11.2%. In the age group 40+ the rate of cardiovascular diseases was even increased to 28% in male and 15% in female skiers [6]. Scheiber et al. recorded blood pressures during skiing on steep slopes with maximum systolic values of more than 200 mmHg in healthy elder recreational skiers [7]. One might speculate that blood pressure peaks in addition to increased intrathoracic pressures predispose to aortic injury. Another matter of discussion is the popular use of the carving technique which produces a higher peak total ground reaction force compared with the parallel ski steering mode or individual technique skiing modes [2]. In the summer seasons only 3/63 (4.8%) of aortic dissections occurred during mountaineering or alpine hiking. Some obvious stress factors like cold temperatures and heavy cold winds are less pronounced in summer. Furthermore one might speculate that the majority of alpine summer hiking is performed with less increase of blood pressure and intrathoracic pressures compared with skiing. 

Half of the skiing-associated aortic dissections happen in January and February. One explanation is that during these two months most skiers are on the slopes. Additionally these two months typically are the coldest ones in the year and thus increase the stress for the body. 

All but one skier who experienced aortic dissection type A had their permanent residency outside our referral area and lived at a median altitude of 170 m. In Tirol there are only two out of 279 villages situated below an altitude of 500 m and 60% of the population lives at an altitude of more than 600 m. Hence the local population is continuously exposed to higher altitudes. Additionally one might speculate that local residents perform their alpine sports activity throughout the year and not only at one time point per year. In skiers aortic dissection happens at a tenfold higher altitude than that of their permanent residence, and there was only a single Tyrolean skier out of the 17 skiers with aortic dissection. This might also be attributable to a physical stress resulting from a long trip from low altitude regions to high altitudes. Klug et al. analyzed all vacationers who were admitted to the University Hospital Innsbruck suffering from acute myocardial infarction (AMI) over a period of 5 winter seasons [8]. In 56% of all cases AMI occurred within the first 2 days of physical exercise, which was mainly alpine skiing. Thus, travel stress plus physical exercise of a not high altitude acclimatized body combined with preexisting cardiovascular diseases may trigger such adverse events. 

In our experience we had only one out of 77 patients in the winter season who suffered from Marfan syndrome, and no other systemic connective tissue disease was present. Hence we could not draw a conclusion with regard to skiing and connective tissue disease.

Maybe the most surprising finding of our study was that the majority of ascending aortic dissections in skiers occurred during “normal” skiing without a trauma. Thus, no patient performed extreme off-piste carving. In the literature scarce information is found on skiing associated aortic dissection. Heller et al. reported 4 cases of traumatic aortic injury (of the descending aorta) following skiing-accidents with a 25% mortality rate [4]. However, no data are available for ascending aortic dissections in skiers. In our study we did not include patients with isolated descending aortic injury, but only aortic dissections involving the ascending aorta. 

In our series skiers were somewhat taller and heavier than nonskiers. One might speculate that increased forces are created by heavier skiers. However, the body mass index was not different between both groups and we are cautious with the interpretation of these data.

The perioperative outcome of skiers with ascending aortic dissection who underwent surgery is favorable with a low mortality. This can in part be explained with the good physical condition of skiers who are practicing sports. 

We conclude that skiing-associated aortic dissection affects persons who perform an unusual physical activity (which they do not perform the rest of the year) and which temporarily increases intrathoracic pressures and systolic blood pressures at an unusual high altitude. Typically skiing-associated aortic dissection happens during recreational skiing without additional trauma. The perioperative course of these patients is favorable with low hospital mortality.

## Figures and Tables

**Table 1:** Demography and preoperative parameters of 77 patients with aortic dissection type A in the winter season (from November to April).

	Nonskiers (*n* = 60)	Skiers (*n* = 17)	*P* value
Age (years)	58 (30–84)	51 (36–79)	0.128
Male gender	46 (76.7%)	15 (88.2%)	0.299
Permanent residency height above sea level (m)	574 (0–1247)	170 (0–853)	<0.001
Weight (kg)	80 (45–110)	90 (68–125)	0.002
Size (cm)	175 (157–191)	180 (172–200)	0.008
Body mass index (kg/m^2^)	26 (18–34)	28 (23–35)	0.102
Marfan syndrome	1 (2%)	0	0.588
Pericardial effusion	16 (26.7%)	4 (23.5%)	0.823
Patient intubated at time of admission	6 (10%)	0 (0%)	0.294
Aortic valve regurgitation II° or more	24 (40%)	5 (29.4%)	0.520
Entry ascending aorta	37 (61.7%)	15 (88.2%)	0.269
Entry aortic arch	12 (20%)	2 (11.8%)	0.269
Entry unknown	11 (18.3%)	0 (0%)	n.a.
Preoperative neurologic deficit	10 (16.7%)	3 (17.6%)	0.940
Coronary artery disease	5 (8.3%)	0 (0%)	0.229
Peripheral vascular disease	2 (3.3%)	0 (0%)	0.476
Arterial hypertension	36 (60%)	8 (47.1%)	0.903

**Table 2:** Intraoperative and postoperative variables of 77 patients with aortic dissection type A in the winter season (from November to April).

	Nonskiers (*n* = 60)	Skiers (*n* = 17)	*P* value
Aortic root replacement	15 (25%)	3 (17.6%)	0.535
Cardiopulmonary bypass time (min)	205 (109–423)	201 (144–600)	0.874
Crossclamp time (min)	126 (45–288)	129 (91–313)	0.822
Hypothermic circulatory arrest	56 (93.3%)	16 (94.1%)	0.357
Circulatory arrest time (min)	40 (5–108)	39 (8–83)	0.630
Lowest body temperature (°C)	18 (14–28)	18.5 (16–32)	0.694
Intensive care unit stay (day)	5 (1–66)	4.5 (1–54)	0.360
Prolonged ventilation requiring tracheostomy	4 (6.7%)	0 (0%)	0.232
Postoperative length of stay (days)	15 (6–66)	9 (5–25)	0.033
Hospital mortality	8 (13.3%)	1 (5.9%)	0.399

## References

[1] Müller E, Schwameder H (2003). Biomechanical aspects of new techniques in alpine skiing and ski-jumping. *Journal of Sports Sciences*.

[2] Scheiber P, Seifert J, Müller E (2012). Relationships between biomechanics and physiology in older, recreational alpine skiers. *Scandinavian Journal of Medicine and Science in Sports*.

[3] Hiratzka LF, Bakris GL, Beckman JA (2010). 2010 ACCF/AHA/AATS/ACR/ASA/SCA/SCAI/SIR/STS/SVM guidelines for the diagnosis and management of patients with Thoracic Aortic Disease: a report of the American College of Cardiology Foundation/American Heart Association Task Force on Practice Guidelines, American Association for Thoracic Surgery, American College of Radiology, American Stroke Association, Society of Cardiovascular Anesthesiologists, Society for Cardiovascular Angiography and Interventions, Society of Interventional Radiology, Society of Thoracic Surgeons, and Society for Vascular Medicine. *Circulation*.

[4] Heller G, Immer FF, Savolainen H, Kraehenbuehl ES, Carrel TP, Schmidli J (2006). Aortic rupture in high-speed skiing crashes. *Journal of Trauma*.

[5] Tirol Werbung GmbH Der Tiroler Tourismus, Zahlen, Daten und Fakten. http://www.tirol.at/.

[6] Faulhaber M, Flatz M, Gatterer H, Schobersberger W, Burtscher M (2007). Prevalence of cardiovascular diseases among alpine skiers and hikers in the Austrian Alps. *High Altitude Medicine and Biology*.

[7] Scheiber P, Krautgasser S, Duvillard SP, Müller E (2009). Physiologic responses of older recreational alpine skiers to different skiing modes. *European Journal of Applied Physiology*.

[8] Klug G, Schenk S, Dörler J (2011). Occurrence of acute myocardial infarction in winter tourists: data from a retrospective questionnaire. *Clinical Research in Cardiology*.

